# Pharmacokinetics, Pharmacodynamics, and Dosing Considerations of Novel β-Lactams and β-Lactam/β-Lactamase Inhibitors in Critically Ill Adult Patients: Focus on Obesity, Augmented Renal Clearance, Renal Replacement Therapies, and Extracorporeal Membrane Oxygenation

**DOI:** 10.3390/jcm11236898

**Published:** 2022-11-22

**Authors:** Dana Bakdach, Reem Elajez, Abdul Rahman Bakdach, Ahmed Awaisu, Gennaro De Pascale, Ali Ait Hssain

**Affiliations:** 1 Department of Clinical Pharmacy, Critical Care, Hamad Medical Corporation, Doha 3050, Qatar; 2 Department of Pharmacy, Infectious Diseases, Hamad Medical Corporation, Doha 3050, Qatar; 3 School of Medicine, Jordan University of Science and Technology, Irbid 3030, Jordan; 4 Clinical Pharmacy and Practice, College of Pharmacy, QU Health, Qatar University, Doha 2713, Qatar; 5 Department of Anesthesiology, Intensive Care and Emergency Medicine, Fondazione Policlinico Universitario A. Gemelli IRCCS, 00168 Rome, Italy; 6 Dipartimento di Scienze Biotecnologiche di Base Cliniche Intensivologiche e Perioperatorie, Universita’ Cattolica del Sacro Cuore, 00168 Rome, Italy; 7 Department of Medicine, Critical Care Services, Hamad Medical Corporation, P.O. Box 305, Doha 3050, Qatar

**Keywords:** novel beta-lactam antibiotics, critical care, augmented renal clearance, extracorporeal membrane oxygenation, renal replacement, obesity, pharmacokinetics, pharmacodynamics

## Abstract

Objective: Dose optimization of novel β-lactam antibiotics (NBLA) has become necessary given the increased prevalence of multidrug-resistant infections in intensive care units coupled with the limited number of available treatment options. Unfortunately, recommended dose regimens of NBLA based on PK/PD indices are not well-defined for critically ill patients presenting with special situations (i.e., obesity, extracorporeal membrane oxygenation (ECMO), augmented renal clearance (ARC), and renal replacement therapies (RRT)). This review aimed to discuss and summarize the available literature on the PK/PD attained indices of NBLA among critically ill patients with special circumstances. Data Sources: PubMed, MEDLINE, Scopus, Google Scholar, and Embase databases were searched for studies published between January 2011 and May 2022. Study selection and data extraction: Articles relevant to NBLA (i.e., ceftolozane/tazobactam, ceftazidime/avibactam, cefiderocol, ceftobiprole, imipenem/relebactam, and meropenem/vaborbactam) were selected. The MeSH terms of “obesity”, “augmented renal clearance”, “renal replacement therapy”, “extracorporeal membrane oxygenation”, “pharmacokinetic”, “pharmacodynamic” “critically ill”, and “intensive care” were used for identification of articles. The search was limited to adult humans’ studies that were published in English. A narrative synthesis of included studies was then conducted accordingly. Data synthesis: Available evidence surrounding the use of NBLA among critically ill patients presenting with special situations was limited by the small sample size of the included studies coupled with high heterogeneity. The PK/PD target attainments of NBLA were reported to be minimally affected by obesity and/or ECMO, whereas the effect of renal functionality (in the form of either ARC or RRT) was more substantial. Conclusion: Critically ill patients presenting with special circumstances might be at risk of altered NBLA pharmacokinetics, particularly in the settings of ARC and RRT. More robust, well-designed trials are still required to define effective dose regimens able to attain therapeutic PK/PD indices of NBLA when utilized in those special scenarios, and thus aid in improving the patients’ outcomes.

## 1. Background

The incidence of deaths owing to sepsis/septic shock in noncardiac patients admitted to intensive care units (ICUs) continues to increase, despite advances in management and personalized treatments Despite advances in management and personalized treatments, mortality rate of infected patients was found to be more than double of that noninfected patients admitted to intensive care units (ICUs) [1,2]. Sepsis-causing multi-drug-resistant (MDR) pathogens, including *Pseudomonasaeruginosa (PsA)*, *Enterobacterspecies*, *methicillin-resistant Staphylococcus aureus*, and *Enterococcus faecium*, have become a remarkable burden, especially for severely ill patients [3,4]. Effective therapy for these patients relies not only on early diagnosis and timely antimicrobial administration, but also on dose-regimen optimization to achieve optimal pharmacokinetic (PK)/pharmacodynamic (PD) targets associated with maximal efficacy.

The interest in the use of novel β-lactam antibiotics (NBLA), which include both novel β-lactam and novel β-lactam/β-lactamase inhibitors (BLBLI) (namely ceftolozane/tazobactam (C/T), ceftazidime/avibactam (CAZ/AVI), cefiderocol, ceftobiprole, imipenem/relebactam (IMI/REL), and meropenem/vaborbactam (MEV)) as alternatives against MDR pathogens among critically ill patients has recently increased. Recommended dosing regimens of antimicrobials are often based on PK studies in healthy volunteers. However, critically ill patients are known to present with various physiological/pathological changes that result in substantial alterations of achieved drug concentrations, along with other key PK parameters [5,6]. Moreover, extracorporeal support (including renal replacement therapies (RRT) and extracorporeal membrane oxygenation (ECMO)) may sometimes be required, further contributing to PK-related variabilities [7,8,9]. Previous researchers utilizing the recommended package insert doses of conventional β-lactams in such heterogeneous populations revealed the risk of both under-/over-exposures depending on the underlying pathology and clinical condition [10,11,12]. Despite growing use in critically ill patients, limited evidence exists describing such variabilities for NBLA. Recently, the adequacy of the current monograph-recommended dosing of three different NBLA in ICU patients was investigated using Monte Carlo simulation [13]. While standard dosing resulted in adequate attainment of a PD index of 40–60% of free drug concentration above MIC (%ƒT > MIC) for CAZ/AVI, C/T, and MEV, such dosing did not attain more aggressive targets (i.e., 100%ƒT > 1–4 × MIC). Although some reviews had recently described altered PK/PD for various NBLA during critical illness [14,15], sub-populations that warrant special considerations including obesity, augmented renal clearance (ARC), RRT modalities, and ECMO were commonly excluded or not comprehensively addressed [14,15,16,17,18]. Hence, this narrative review seeks to [1] summarize the evidence for the effects of common ICU circumstances (including ECMO, ARC, RRT, and obesity) on the PK of NBLA, and [2] evaluate whether the utilized dosing regimens were adequate in achieving the required PK/PD indices.

## 2. Methods

PubMed, MEDLINE, Scopus, Google Scholar, and Embase databases were searched for studies published between January 2011 and May 2022 (to focus on NBLA approved during the last decade). Searched keywords included C/T, CAZ/AVI, cefiderocol, ceftobiprole, IMI/REL, and MEV. The following terms were used in combination with the keywords listed above: “obesity”, “augmented renal clearance”, “renal replacement therapy”, “extracorporeal membrane oxygenation”, “pharmacokinetic”, “pharmacodynamic”, “critically ill”, and “intensive care”. The search was limited to adult humans and articles published in English. Two authors (A. B. and R. E.) independently performed the systematic search. Disagreements were resolved by referring to a third author (D. B.). The inclusion of articles in this review was guided by the Population, Intervention, Comparator, Outcome (PICO) framework outlined in detail in Table 1. No additional analyses of the risk of bias were performed since the intention was purely descriptive narration.

## 3. Results

**Figure 1:**
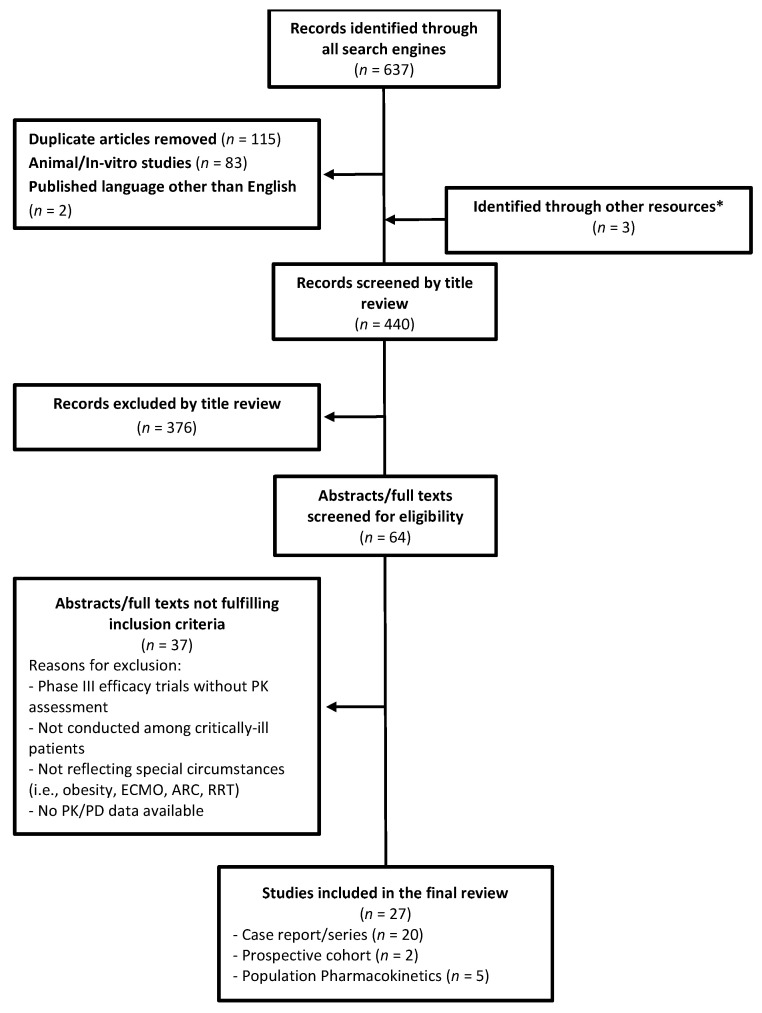
Flow diagram of the study selection. * Three abstracts/posters were indentified through searching and were included in the review since pertinent data regarding PK/PD and target attainments in critically-ill patients were available.

The initial search yielded 637 articles. After removing duplicates and applying limitations stated above, 64 full-text articles were obtained. Of these, 27 fulfilled the PICO outlined in Table 1 and thus were included in this review. A detailed overview of the selection process is illustrated in Figure 1.

### 3.1. Altered PK and Associated PD Targets

Physiologic alterations encountered in critically ill populations have been implicated in influencing drugs’ PK, specifically the two primary PK parameters: volume of distribution (Vd) and clearance (CL). 

Endothelial dysfunction, capillary leakage, and fluid resuscitation, along with other factors result in increased Vd of hydrophilic antimicrobials. Clearance, on the other hand, is often determined by the properties of the drugs, with hydrophilic antimicrobials being principally cleared through the renal pathway. Both extremes of renal clearance (i.e., ARC/renal failure) are encountered in ICU and can thus have profound effects on the elimination of hydrophilic renally-eliminated antimicrobials. Hypoalbuminemia, as an acute-phase reactant, is commonly seen in critically ill patients and can potentially further impact the PK of antimicrobials, especially the highly protein-bounded. The increased free/unbound concentrations results in increased Vd and can be coupled with decreased overall exposure of the renally-cleared antimicrobials, as more unbound fraction is gaining access to the nephrons for elimination [19,20,21,22]. A detailed description of different sources of PK variabilities encountered during critical-illness is beyond the scope of this paper, and readers are referred to the literature for further details [23,24,25].

Given their physicochemical and PK properties, namely hydrophilicity and renal CL, NBLA are therefore considered susceptible to exposure discrepancies when used among critically ill patients [26,27,28]. 

Pharmacodynamically, like conventional β-lactams, the %ƒT > MIC has been described as the optimal PK/PD index associated with the efficacy of NBLA. However, the optimal percentage to target is still controversial [29]. Preclinical studies of conventional β-lactams suggested that approximately 1–2 log_10_ reductions of colony-forming units (CFU) can be achieved with <100%ƒT > MIC, depending on the used antimicrobial (i.e., 40%, 50–60%, and 50–70%ƒT > MIC for carbapenems, penicillins, and cephalosporins respectively) [30]. Although these animal/in-vitro derived targets were replicated in human studies, promising outcomes were not always consistent and some suggested higher than 40–70%ƒT > MIC might sometimes be required for favorable outcomes, especially among severely sick patients [30,31]. For instance, the Defining Antibiotic Levels in ICU(DALI) trial described significant exposure variabilities of conventional β-lactams when used in ICU patients, with one-fifth of the included cohort failing to achieve the most conservative PD index of 50%ƒT > MIC. On the contrary, higher odds of favorable clinical outcomes were seen amongst patients achieving 50%ƒT > MIC and 100%ƒT > MIC targets (OR 1.02, 1.56 respectively; *p* < 0.03) [32]. Likewise, McKinnon et al., compared the PD of ceftazidime and cefepime and observed significantly higher rates of bacteriological eradication and clinical cure among patients achieving 100%ƒT > MIC as opposed to <100%ƒT > MIC [33]. Similar conclusion was recently reported with meropenem and piperacillin-tazobactam, with faster infection resolution being observed among patients attaining 100%ƒT *>* MIC target [34].

Although the utilization of conventional PD thresholds (i.e., 40–60%ƒT > MIC) could be argued for NBLA, especially when considering the concentrations of BLIs, for the most part, BLIs by their own are inactive against invading pathogens [30]. The PD targets of such inhibitors (i.e., threshold of concentration (C_T_) for avibactam/tazobactam and 24 h area under the free concentration–time curve (*f*AUC_24_/MIC) for vaborbactam/relebactam) reflect the required concentrations to restore β-lactam activity, thus leaving PD targets of β-lactam backbone consistent with the PD targets described above for the severely sick population [29,35]. 

More aggressive indices (i.e., 100%ƒT > 4–8 × MIC) were recently advocated among critically ill patients (especially empirically upon initiation when the pathogen/MIC are still unknown) to improve the likelihood of favorable outcomes and prevent resistance development, a detrimental consequence frequently encountered in the ICU population [34,36,37,38,39,40,41,42]. However, given the limited available data, coupled with safety concerns of increased adverse outcomes/toxicities associated with such higher trough concentrations, this target might be more appropriately tailored to individualized situations only [34,36,39,43,44,45].

Hence, in this review, we stated the PK/PD indices that were targeted by the authors of each included study, and then compared the adequacy of the used regimen in attaining the aggressive target of 100%ƒT > MIC.

The leaflet-derived PK parameters along with conventional murine/in vitro-derived PD targets of NBLA included in this review are summarized in Supplementary Materials (Table S1) for reference. Table 2 summarizes the included studies in this review, along with their utilized dosage regimens and attained PD indices. 

### 3.2. Obesity

Antimicrobial dosing for critically ill obese patients is challenging. Different pathophysiologic alterations seen in obesity can result in additive effects of altered PK including increased cardiac output, lean/fat masses, kidney size, and renal blood flow [39,46]. For hydrophilic antimicrobials, increased lean mass, coupled with increased renal CL translate to more PK variations. Different PK studies of conventional β-lactams confirmed altered PK in obese/morbidly-obese patients compared to non-obese population [44,47,48]. However, whether the effect of altered PK is significant enough to warrant dosage adjustment is still debatable [48,49]. 

Cojutti and colleagues [50] described the effect of obesity on PK/PD of ceftobiprole when used as an add-on to daptomycin against methicillin-resistant *Staphylococcus epidermidis* bacteremia in a morbidly obese critically ill patient (BMI 51.2 kg/m^2^). The utilization of standard dosing (0.5 g Q8h) as extended infusion (E.I) over 4 h (vs. recommended 2 h) resulted in 100%ƒT > MIC_≤2 mg/L_ attainment. However, more frequent administrations (Q6h) coupled with E.I were required to attain a more aggressive index (100%ƒT > 3 × MIC) and resulted in a favorable clinical outcome. Unfortunately, only maximum and minimum concentrations (C_max_, C_min_ respectively) were reported in this case report, and thus the effects of obesity on other PK parameters could not be determined. Similarly, C/T use at the manufacturer’s recommended regimen resulted in an adequate exposure (100%ƒT > MIC) when used to treat ventilator-associated pneumonia in a morbidly obese patient, with higher targets (100%ƒT > 4 × MIC) being achieved using the continuous infusion (C.I) technique [51]. In both cases, the dose required to attain the conventional PD index (i.e., 30–50% and 40%ƒT > MIC for ceftobiprole and C/T respectively) was not investigated. No studies were found describing the effect of obesity on PK of CAZ/AVI, cefiderocol, IMI/REL, and MEV in critically ill patients. 

In summary, limited data exist to guide dosing of NBLA in critically ill morbidly/obese patients, and interpretation of antimicrobial exposure is thus difficult, given the variabilities that exist in such populations. More research is still required, and therapeutic drug monitoring (TDM) might be warranted to guide therapy across such groups.

### 3.3. Extra-Corporeal Membrane Oxygenation (ECMO)

ECMO has been believed to have various effects on PK of antimicrobials, including altered protein binding, increased Vd (secondary to fluid boluses, transfusion requirements, drug sequestration into ECMO circuits, etc.) and altered CL, with lipophilic highly protein-bound drugs being mostly affected [7,52]. These observations were mainly extrapolated from old neonatal PK studies [53]. Nevertheless, despite the hydrophilicity and limited protein-binding of NBLA, the additive effects of critical-illness and ECMO might correlate with altered PK, and hence result in exposure variabilities.

The effects of ECMO on NBLA’s PK/PD were documented in four case reports/series: two with C/T [54,55] and two for cefiderocol [56,57].

Arena et al. utilized C/T in treating persistent *PsA* pneumonia in a patient requiring venoarterial ECMO (VA-ECMO) [54]. The standard dosage (3 g Q8h) was considered sufficient for both the conventional target and the aggressive target of 100%ƒT > MIC. Notably, the authors observed lower C_max_ and C_min_ during the last 2 days of therapy and attributed it to increased Vd (positive fluid balance) and/or enhanced renal CL.

In the other case, C/T was used as a part of chemoprophylaxis in a cystic fibrosis patient requiring ECMO post-lung transplantation [55]. RRT via continuous venovenous hemodiafiltration (CVVHDF) was also required for this patient, owing to acute kidney injury (AKI). When administered as an unadjusted regimen (i.e., regular manufacturer dosing irrespective of AKI/RRT modality), 100%ƒT > 4 × MIC was attained for ceftolozane. It was concluded that ECMO did not require dosing modifications; rather, given the incidence of AKI, reduced dosing might have been adequate. 

Comparable with C/T, minimal effects on the PK of cefiderocol among two ECMO case-series [56,57] with adequate concentrations (i.e., 100%ƒT > MIC) were achieved using the regular manufacturer’s regimen.

In summary, based on the available human data, ECMO seemed to have minimal effects on NBLA. However, given the scarcity of reports, the use of CVVHDF in one patient, and keeping in mind that most reports were utilizing VA-ECMO [which might result in different physiological effects as opposed to venovenous ECMO (VV-ECMO)], more data are still required to confirm the observation. Additionally, no studies were found reporting the effect of ECMO on the PK of CAZ/AVI, ceftobiprole, IMI/REL, or MEV. 

### 3.4. Augmented Renal Clearance (ARC)

ARC is a phenomenon frequently encountered across the ICU population secondary to fluid resuscitation, coupled with enhanced cardiac output, leading to amplified renal perfusion. ARC (defined as creatinine clearance (CrCL) > 130 mL/min/1.73 m^2^) has been linked to lower plasma drug concentrations of renally cleared antimicrobials and worse clinical outcomes [58,59,60]. Given the hydrophilicity and predominant renal clearance of NBLA, ARC may increase the risk of therapeutic failure and/or drug resistance. A total of six studies were retrieved describing the effects of ARC on PK of C/T [61,62], CAZ/AVI [26], IMI/REL [63] and ceftobiprole [50,64].

Sime et al. analyzed the PK of C/T using data from 12 ICU patients receiving either 1.5 g or 3 g Q8h regimens and performed Monte Carlo simulations to predict optimal regimens for critically ill patients with preserved kidney functionality, including those with ARC [61]. When utilized empirically (i.e., aiming for 40%ƒT > MIC_≤64 mg/L_ to cover *PsA*), a 1.5 g-regimen was found inadequate among the ARC cohort, and a 3 g regimen was required. Nevertheless, when a more aggressive index was targeted (i.e., 100%ƒT > MIC_≤64 mg/L_), the 3 g intermittent regimen was not satisfactory, and 1.5 g loading over 30 min followed by 4.5 g C.I (over 24 h) was suggested instead. In contrast, directed therapy against MIC_≤4 mg/L_ was adequately achieved with 1.5 g and 3 g intermittent regimens when aiming for 40% and 100%ƒT > MIC_≤4 mg/L_ respectively. Remarkably, the suggested empiric regimens were selected, aiming for 100%ƒT > 4 × MIC_≤16 mg/L_, which is not routinely targeted in clinical practice nor representative given the sensitivity breakpoint of ceftolozane (≤4, 2 mg/L for *PsA* and *Enterobacterales*, respectively [65]).

In early 2021, the results of a phase I PK study of C/T among critically ill patients with confirmed ARC (using the 8 h urine collection method) were published [62]. The mean Vd was 1.5-fold higher in critically ill patients with ARC with a resultant lower C_max_ than that of retrospective healthy cohorts. A single dose of 3 g was considered sufficient to achieve 40%ƒT > MIC_≤4 mg/L_ and almost half of the patients were able to attain 100%ƒT > MIC_≤4 mg/L_ for ceftolozane with the same dose. It is noteworthy that, this was a single-dose PK assessment and thus might not reflect effects of accumulation/repeated administrations. 

The PK of ceftobiprole use in ARC was investigated in a multicenter, open-label, and non-randomized trial [64]. The systemic CL of ceftobiprole in patients with CrCL > 150 mL/min was two-fold higher than that in patients with normal/slightly decreased CrCL. As part of the study protocol, patients received 1 g Q8h as E.I over 4 h (as opposed to 0.5 g over 2 h, according to the manufacturer’s dosing). This resulted in attaining 100%ƒT > MIC≤_4 mg/L_. When extrapolated to the manufacturer’s recommended dosing of 0.5 g, only E.I over 4 h allowed the attainment of 100%ƒT > MIC [66]. Likewise, administration of 0.5 g by Cojutti et al. [50] was sufficient to attain 100%ƒT > MIC_≤2 mg/L_ when E.I over 4 h was utilized.

Serum levels of CAZ/AVI following administration of the recommended dose (2.5 g Q8h over 2 h)were used in a phase 4 study to simulate effects of CrCL on CAZ/AVI’s PK among critically ill patients [26]. Despite a two-fold increase in Vd of CAZ compared to healthy volunteers, the probability of target attainment(PTA) was successfully increased in patients with ARC when aiming for 50%ƒT > MIC_≤16 mg/L_of CAZ. Higher targets (i.e., 100%ƒT > MIC_≤16 mg/L_) were not investigated, and thus the adequacy of CAZ/AVI regular dosing among ARC patients when aiming for aggressive targets is yet to be defined. 

The effects of ARC on IMI/REL’s PK were recently presented by Fratoni and colleagues [63]. Compared to healthy volunteers, the researchers observed an increased CL of IMI/REL. However, regular dosing (1.25 g over 30 min) was found adequate in providing IMI exposures > 40%ƒT > MIC (range: 40–90%). It is worth noting that this was a single-dose PK study, thus repeated administrations’ effects were not investigated, nor was an aggressive target (100%ƒT > MIC) achieved by any of the included cohorts.

Cefiderocol is the only NBLA that has a manufacturer recommendation for ARC. Such a recommendation was initially based on simulations using healthy volunteers’ concentrations, as opposed to critically ill patients [67,68]. However, using plasma concentrations of patients with ARC including patients from APEKS-NP (with 70% of the cefiderocol group being in ICU at time of randomization) and CREDIBLE-CR (with more than 50% of the cefiderocol group being in ICU at time of randomization), Kawaguchi and colleagues reported that adequacy of such a regimen (i.e., 2g Q6h each administered as E.I over 3 h), with its ability to attain 100%*f*T > MIC for MIC ≤ 4 mg/L [69,70,71]. Similarly, although MEVs’ PKs were not described for an ARC setting, extrapolation from meropenem use among ICU patients with ARC might be considered. Different reports have highlighted reduced meropenem concentrations in such settings, coupled with required regimens’ modifications to attain a PD target [60,72,73,74]. Moreover, keeping in mind the predominant renal CL of vaborbactam, studies are therefore required to characterize ARC effects on MEV in terms of not only altered PK/required dosage adjustments, but also retained efficacy. 

In summary, ARC appeared to result in a disturbed PK of NBLA. Modified dosing regimens (including higher dosing, extended/or C.I) coupled with TDM might be warranted, especially when aggressive targets (i.e., 100%*f*T > MIC or higher) are required. 

### 3.5. Renal Replacement Therapy

AKI is commonly encountered in ICU patients, resulting in the accumulation of renally cleared drugs, including NBLA, and can result in potential toxicities. Nevertheless, many of these patients require extracorporeal renal support (i.e., RRT) in form of either prolonged intermittent renal replacement therapy (PIRRT) or continuous renal replacement therapy (CRRT), based on the clinical scenario [75]. The differences in the used modalities (e.g., effluent flow rate, filter type, renal replacement method, etc.), patient’s residual kidney functionality, and the disturbed PK owing to critical illness may render the dosing of NBLA in such settings challenging [14,15]. 

#### 3.5.1. Prolonged Intermittent Renal Replacement Therapy (PIRRT)

A case report described effects of PIRRT on C/T’s PK when used in a critically ill patient with MDR *PsA* (MIC: 4 mg/L) [76]. The patient received a loading dose of 0.75 g C/T (over 1.5 h), followed by 0.15 g Q8 h and 0.75 g Q12 h on non-PIRRT and PIRRT days respectively. Even though higher amounts of ceftolozane and tazobactam were removed during PIRRT than non-PIRRT days (>20× difference in overall CL), the administration of the aforementioned regimen during and immediately after PIRRT replenished the lost amount, and maintained concentrations above MIC for the entire therapy duration (both 40%, 100%ƒT > MIC for ceftolozane). 

The effects of PIRRT on CAZ/AVI, ceftobiprole, cefiderocol, IMI/REL, and MEV when used among ICU patients were not reported.

In summary, little data exist regarding the effects of PIRRT on the PK of NBLA. Until more data are available, therapies might better be guided by TDM to ensure adequacy. 

#### 3.5.2. Continuous Renal Replacement Therapy (CRRT)

Gatti and Pea have recently described the effects of various CRRT modalities on PK of NBLA [14]. Relevant studies provided in reviews by Gatti and Pea [14,15], along with several other studies, are highlighted in Table 2. In summary, NBLA’s PK is influenced by CRRT type (continuous venovenous hemofiltration (CVVH), continuous venovenous hemodialysis (CVVHD), and CVVHDF), flow rates, and dilutional fluids. The PK is further affected by residual kidney functionality, which can impact its ability to attain the desired PD index. The results from different reported studies (highlighted in Table 2) suggest that higher doses and/or longer infusions might be necessary in cases of residual kidney function (as compared to anuric states), or when higher PK/PD indices are targeted, especially with highly-resistant pathogens. For a more detailed discussion on the effects of CRRT, readers are advised to refer to Gatti and Pea’s review [14,15]. 

Based on the limited available evidence, Table 3 briefly provides our suggested initial regimens of different NBLA based on PK/PD targets (i.e., in-vitro derived vs. 100%ƒT > MIC targets) when used among critically ill patients with special scenarios. However, given that most of the reported data were based on case reports/series, those recommendations might be considered as initial dosing regimens. TDM might still be warranted especially among unstudied scenarios or when other PK/PD indices are targeted (e.g., 100%*f*T > 4–8 × MIC).

**Table 2:** Novel β-lactam antibiotics utilized dosing regimens and PK/PD target attained of in the included studies.

Reference	Study Design (# of Patients)	Source of Infection	Pathogen/ MIC (mg/L)	PK/PD Target Aimed by Investigators	Dose Administered	Patient(s) Creatinine Clearance (mL/min)/Urine Output (mL/Day)	Studied Scenario(s)				PK/PD Target Achieved with Given Regimen	Clinical Outcome
							ARC	RRT	ECMO	Obesity		
**Ceftolozane/Tazobactam**												
Kuti et al. [77]	Case Report (1)	VAP	*P. aeruginosa* MIC: 0.75/4	100%ƒT > MIC	3 g Q8h (**over 1 h**)	<80 mL/day		CVVHDF			100%ƒT > MIC	Clinical cure
Bremmer et al. [78]	Case Report (1)	BSI, VAP, Osteomyelitis	*P. aeruginosa* MIC: 2	100%ƒT > MIC	3 g Q8h (**over 1 h**)	<50 mL/day		CVVHDF			100%ƒT > MIC_≤8 mg/L_	Clinical cure *
Carbonell et al. [79]	Case Report (1)	CRBSI	*P. aeruginosa* MIC: NR	100%ƒT > 4 × MIC_≤4 mg/L_	3 g Q8h (**over 3 h**)	NR		CVVHDF (+oXiris filter) + MARS			100%ƒT > 4 × MIC_≤4 mg/L_	Clinical failure
Aguilar et al. [80]	Case Report (1)	cIAI	NR	100%ƒT > MIC_≤8 mg/L_	3 g Q8h (**over 1 h**)	0 mL/day		CVVHD			100%ƒT > MIC_≤8 mg/L_	Clinical cure
Oliver et al. [81]	Case Report (1)	Osteomyelitis	*P. aeruginosa* MIC: 1.5	100%ƒT > MIC	1.5 g Q8h (**over 4 h**)	NR		CVVH			100%ƒT > 8 × MIC_≤4 mg/L_	Clinical cure
Mahmoud et al. [51]	Case Report (1)	VAP	*P. aeruginosa* MIC: 2/4	100%ƒT > MIC 100%ƒT > 4 × MIC_≤2 mg/L_	3 g Q8h (**over 1 h**); then changed to 9 g/24 h (**as C.I**)	0 mL/day		CVVHDF		BMI 54.5 kg/m^2^	100%ƒT > MIC and 100%ƒT > 4 × MIC_≤2 mg/L_	NR
Sime et al. [82]	PK population Study (6)	Unknown (*n* = 1) Lung (*n* = 1) BSI (*n* = 2) BSI + Lung (*n* = 2)	Polymicrobial including *P. aeruginosa*, *S. maltophilia*, *S. marcescens*, *K. pneumoniae and others*; MIC: NR	**Ceftolozane**: 40%ƒT > MIC_≤4 mg/L_ Simulations done for 60%, 100%ƒT > MIC **Tazobactam**: Simulations done for 20%ƒT > C_T:1 mg/L_ 50%ƒT > C_T:2 mg/L_100%ƒT > C_T:4 mg/L_	1.5 g Q8h (**over 1 h**)	NR		CVVHDF			**Empiric therapy** (*first 24 hr; covering EUCASTP. aeruginosa sensitivity breakpoint of 4 mg/L*): - 40%ƒT > MIC: 0.75 g Q8h - 100%ƒT > MIC 1.5 g Q8h (**over 1 h**) or 3 g Q8h (**over 1 h**) or 3 g LD then 0.75 g Q8h (**over 1 h**) - 100%ƒT > MIC (assuming empirically coverage of up to MIC_≤64 mg/L_): 3 g LD plus 9 g/24 h C.I **Targeted therapy** (*after 24 h and known MIC≤4 mg/L*): - 40%ƒT > MIC: 0.75 g q8h (**over 1 h**) (lower dosing can be theoretically possible) - 100%ƒT > MIC: 0.75g Q8 (**over 1 h**) (*Note: 0.75 g Q8h achieved adequate Tazobactam targets of 20%ƒT* > C_T:1 mg/L_*and 50%ƒT* > C_T:2 mg/L_	NA
Rawlins et al. [76]	Case Report (1)	Osteomyelitis	*P. aeruginosa* MIC: 4	42%ƒT > MIC	**Off dialysis days**: LD 0.75 g (**over 1.5 h**), then 0.15 g Q8h **Dialysis days**: 0.75 g Q12h	NR		PIRRT			100%ƒT > MIC	NR
Sime et al. [61]	PK population Study (12)	BSI (*n* = 2), CNS abscess (*n* = 3), CIAI (*n* = 3), UTI (*n* = 1), Pneumonia (*n* = 9), Vascular access (*n* = 1)	Multiple organisms MIC: NR	**Ceftolozane**: Simulations done for 40%, 60%, 100%ƒT > MIC **Tazobactam**: Simulations done for 20%ƒT > C_T:1 mg/L_	1.5 g Q8h (**over 1 h**) and 3 g Q8h (**over 1 h**)	Median: 107 mL/min/1.73 m^2^ IQR: 74–145 mL/min/1.73 m^2^	Simulated for Crcl > 140 and >180 mL/min/1.73 m^2^				**Empiric therapy (MIC unknown; covering emprically for *P. aeruginosa* with MIC up to 64):** **- 40%ƒT > MIC_≤64 mg/L_**: 3 g Q8h (over 1 h) **-100%ƒT > MIC**≤_64 mg/L_: 1.5 g LD then 4.5 g/24 h C.I **Targeted therapy (Known MIC≤4 mg/L):** **-40%ƒT > MIC**≤_4 mg/L_: 1.5-g q8h (over 1 h) -**100%ƒT > MIC**≤_4 mg/L_: 3 g q8h or 1.5 g LD then 4.5 g/24 h C.I **100%ƒT > 4–5 × MIC**≤_4 mg/L_: 3 g LD then 9 g/24 h C.I *(Note: all doses achieved adequate Tazobactam targets of 20%ƒT* > C_T:1 mg/L_	NA
Nicolau et al. [62]	Phase 1, prospective study (11)	NR	NR	**Ceftolozane**: 39%ƒT > MIC_≤4 mg/L_ **Tazobactam**: 20%ƒT > C_T:1 mg/L_	3 g (single dose; **over 1 h**)	>130 mL/min	CrCL > 130 mL/min				**Ceftolozane**: 86%ƒT > MIC≤_4 mg/L_ (*did not achieve 100%ƒT > MIC≤_4 mg/L_*) **Tazobactam**: 55%ƒT > C_T:1 mg/L_	NA
Arena et al. [54]	Case Report (1)	Nosocomial pneumonia	*P. aeruginosa* MIC: 4	**Ceftolozane** 60%ƒT > MIC	3 g Q8h (**over 1 h**)	Day 1: ~90 mL/min, Day 4: ~150 mL/min			VA-ECMO		**Ceftolozane** 100%ƒT > MIC and 100%ƒT > 3.9 × MIC **Tazobactam** *100%ƒT* > C_T:1 mg/L_	Clinical cure
Argudo et al. [55]	Case Report (1)	Post lung transplant prophylaxis (History of CF with XDR-PA colonization)	*P. aeruginosa* MIC: 1.5	100%ƒT > MIC	3 g Q8h (**over 1 h**)	0 mL/day		CVVHDF	VA-ECMO		100%ƒT > MIC and 100%ƒT > 25 × MIC≤_1.5 mg/L_	NR
**Ceftazidime/Avibactam**												
Stein et al. [26]	Phase 4 PK analysis (10)	Pneumonia (*n* = 9) Urosepsis (*n* = 1)	Different Enterobacteriales MIC: NR	**CAZ**: 50%ƒT > MIC **AVI**: 50%ƒT > C_T:1 mg/L_	2.5 g Q8h (**over 2 h**)	Mean: 103 mL/min (range: 47–190 mL/min)	2 patients had ARC; simulations done for CrCL 130–190 mL/min				50%ƒT > MIC≤_16 mg/L_ (based on Monte Carlo simulations) *(100%ƒT > MIC was not investigated)*	NA
Wenzler et al. [83]	Case Report (1)	BSI	*P. aeruginosa* MIC: 6	**CAZ**: 100%ƒT > MIC_≤6 mg/L_ **AVI**: 100%ƒT > C_T:1 mg/L_	1.25 g Q8h (**over 2 h**)	NR		CVVH			**CAZ**: 100%ƒT > MIC 100%ƒT > 4 × MIC **AVI**: 100% fT > C_T:7 mg/L_	Death
Soukup et al. [84]	Case Report (1)	Pneumonia	*P. aeruginosa* MIC: 8	**CAZ**:100%ƒT > 4 × MICfor MIC≤ **AVI**: 100%ƒT > C_T:1 mg/L_	2.5 g Q8h (**over 2 h**)	<100 mL/day		CVVHDF			**CAZ**: 100%ƒT > 4 × MIC **AVI**: 100% fT > C_T:1 mg/L_	Clinical cure
Kline et al. [85]	Case series (6)	NR	NR	**CAZ**: 100%ƒT > 1 and 4 × MIC_≤8 mg/L_ **AVI**: 100%ƒT > C_T:1and2_._5 mg/L_	2.5 g Q8h (**Infusion duration NR**)	NR		CVVHDF			**CAZ**: - 90% of patients achieved 100%ƒT > MIC, - 55% achieved 100%ƒT > 4MIC **AVI**: - 100% of pts achieved 100%ƒT > C_T:1 mg/L_ - 80% of pts achieved 100%ƒT > C_T:2_._5 mg/L_	NR
Zhang et al. [86]	Case report (1)	Pneumonia	K. pneumoniae	CAZ: 100%ƒT > 4 × MIC_≤8 mg/L_	2.5 g Q12h (**over 2 h**)	30 mL/day		CVVHD			100%ƒT > 4 × MIC≤_8 mg/L_	Death *
**Cefiderocol**												
Kobic et al. [87]	Case report (1)	Pneumonia and BSI	*P. aeruginosa* MIC: 4	82%ƒT > MIC_≤4 mg/L_	2 g Q8h (**over 3 h**)	0 mL/day		CVVHDF			>90%ƒT > MIC_4–8 mg/L_ for anuric, residual CrCL 11 and residual CrCL 27 mL/min	Clinical cure
					1.5 g Q12h (**over 3 h**) ***extrapolated***						>**82%ƒT > MIC_≤4 mg/L_** for anuric, or residual CrCL 11 and residual CrCL 27 mL/min **97%ƒT > MIC_≤8 mg/L_**for anuric, 81%fT > MIC_≤8 mg/L_for residual CrCL 11, **65%fT > MIC_≤8 mg/L_**for residual CrCL = 27 mL/min	
König et al. [56]	Case series (5)	Pneumonia and/or BSI	*P. aeruginosa* *A. baumanii* MIC: *0.125*–*0.5*	75%ƒT > MIC_<2 mg/L_	2 g Q8h (**over 3 h**, 4 cases) 1 g Q8h (**over 3 h**, 1 case)	-**Pt 1**: 10 mL/min -**Pt 2**: Day 1: 67 mL/min, Day 7: 22 mL/min -**Pt 3**: >80 mL/min -**Pt 4**: 28 mL/min -**Pt 5**: NR		CVVHD (3 cases)	VA-ECMO (2 cases)		- 100%ƒT > MIC_≤2 mg/L_ - 100%ƒT > 4 × MIC_≤2 mg/L_	3 patients with microbiological cure, 2 patients died
Fratoni et al. [27]	Case report (1)	Bacteremia and pneumonia	*ESBL—Escherichia coli* bacteremia MIC: NR *S. maltophilia* pneumonia MIC: 0.125	100%ƒT > MIC	2 g Q12h (**over 3 h**)	0 mL/min		CVVHDF **Note:** Pt had 20% protein binding as opposed to 58% of package insert			**100%ƒT > MIC**_≤16 mg/L_ for protein binding of 20% and no residual kidney function **>92%ƒT > MIC**_≤4 mg/L_ for all scenarios of anuria, residual CrCL of 15–30 mL/min and protein binding of 20–58%	Clinical cure *
Wenzler et al. [88]	Population PK (9)	nosocomial pneumonia (*n* = 3) and carbapenem-resistant Gram-negative infection (*n* = 6)		75%ƒT > MIC_0.25–16 mg/L_	Patient received: 1 g q12h (**over 3 h**) for CVVH 1.5 g q12h (**over 3 h**) for CVVHD and CVVHDF	Modeling based on assumption of minimal residual kidney functionality		CVVH CVVHD CVVHDF			>90% PTA of 75%ƒT > MIC_≤4 mg/L_ at effluent flow rates from 0.5 to 5 L/h **Simulated regimens required (each infused over 3 h)**: - 1.5 g every 12 h for effluent flow rate ≤ 2 L/h - 2 g every 12 h for effluent flow rate 2.1–3 L/h - 1.5 g every 8 h for effluent flow rate 3.1–4 L/h - 2 g every 8 h for effluent flow rate ≥4.1 L/h	NR
Gatti et al. [57]	Case series (5/13 with special scenario)	Pneumonia and/or BSI	XDR-Acinetobacter baumannii MIC: 0.5–1	*Optimal if f*C_min_/MIC ≥ 4 (100%ƒT > 4 × MIC) *Quasi optimal if f*C_min_/MIC 1–4 (100%ƒT > 1–4 × MIC)	2 g q8h (**over 3 h**) for 4 pts [*1 case received 1.5 g q8h (**over 3 h**)*]	NR		CVVHDF (2 cases)	ECMO (4 cases)		All patients achieved 100% *f*T > MIC (*f*Cmin/MIC > 1) (3*/5 achieved target of fCmin/MIC > 4 (i.e., 100%ƒT > 4 × MIC*))	3/5 documented microbiological eradication
**Ceftobiprole**												
Torres et al. [64,66]	Multi-center, open-labelled non-RCT (31)	NR	NR	ƒT > MIC_≤4 mg/L_(in hours)	CrCL > 80 mL/min: 1 g Q8h (**over 4 h**)	>80 mL/min	CrCL > 150 mL/min were included (6 patients)				- CrCL 80–150 mL/min: 13.2 h - CrCL > 150 mL/min: 10.8 h *(=100%ƒT > MIC_≤4__mg/L_*	NR
											**Extrapolated dosing of 0.5 g Q8h (over 4 h)** 100%ƒT > MIC_≤4 mg/L_ (*PTA: 100% if CrCL 80–150 mL/min* vs. *~90% if CrCL > 150 mL/min*)	
Cojutti et al. [50]	Case report (1)	BSI	*Methicillin Resistant Staphylococcus epidermidis (MRSE)* MIC: 2	C_min_/MIC: 1–4 (i.e., 100%*f*T > 1–4 × MIC)	0.5 g Q8h (**over 4 h**)	>120 mL/min	CrCL > 120 mL/min			BMI 51.2 kg/m^2^	Cmin/MIC 2.85 (=100%ƒT > 2.4 × MIC_≤2 mg/L_	Clinical cure
				C_min_/MIC: 1–4 (i.e., 100%*f*T > 1–4 × MIC) *Aiming for higher end of the range*)	0.5 g Q6h (**over 4 h**)						Cmin/MIC 3.19 (=100%ƒT > 2.7 × MIC_≤2 mg/L_)	
Cojutti et al. [89]	Case report (1)	Pneumonia	Empirically covering *Methicillin-resistant Staphylococcus aureus (MRSA)* MIC: NR	C_min_/MIC: 1–4	0.25 g Q12h (**over 2 h**)	10 mL/min/1.73 m^2^		CVVHDF			C_min_/MIC 2.12 100%ƒT > 1.8 MIC_≤2 mg/L_	Clinical cure *
**Imipenem/Relebactam**												
Fratoni et al. [63]	Prospective PK analysis (5)	NR	NR MIC: 2	- IMI 30%*f*T > MIC) - REL (*f*AUC:MIC 18) For MIC ≤ 2 mg/L	1.25 g once (**over 30 min**)	> 130 mL/min	CrCL > 130 mL/min				- IMI 40–90%ƒT > MIC≤_2 mg/L_ - REL fAUC:MIC ranged 22.6–59.0	
**Meropenem/Vaborbactam**												
Kufel et al. [90]	Case report (1)	Periprosthetic hip joint infection	*K. pneumoniae* MIC: 0.094/8	100%ƒT > MIC	2 g Q8h (**over 3 h**)	0 mL/day		CVVHD			100%ƒT > MIC (for MIC 4/8 mg/L of Meropenem/vaborbactam respectively)	Clinical failure

**MIC**: minimum inhibitory concentration, **ARC**: augmented renal clearance, **RRT**: renal replacement therapy, **ECMO**: extracorporeal membrane oxygenation, **BSI**: bloodstream infection, **CNS**: central nervous system, **VAP**: ventilator-associated pneumonia, **cIAI**: complicated intra-abdominal infection, **CRBSI**: catheter-related bloodstream infection, **CVVH**: continuous veno-venous hemofiltration, **CVVHD**: continuous venovenous hemodialysis, **CVVHDF**: continuous venovenous hemodiafiltration, **PIRRT**: prolonged intermittent renal replacement therapy, **BMI**: body mass index, **VA-ECMO**: venoarterial extracorporeal membrane oxygenation, **NR**: not reported, **NA**: not applicable, **MARS**: molecular adsorbent-recirculating-system, **C.I**: continuous infusion, **LD**: loading dose, **CF**: cystic fibrosis, **XDR**: extensively drug-resistant, **ESBL**: Extended Spectrum B-Lactamase, **Pt**: patient, **AUC**: area under the concentration time curve, **%ƒT > MIC**: percentage of free drug concentration above MIC, **Cmin**: minimal concentration, **PK/PD**: pharmacokinetic/pharmacodynamic, **CrCL**: creatinine clearance, **Q**x**h**: every x hours, **IMI/REL**: imipenem/relebactam, **CAZ/AVI**: ceftazidime/avibactam. * Patient death was considered non-infection related. Although ARC is commonly defined as >130 mL/min/1.73 m^2^, different studies used different cut-offs ranging from >120 to >160 mL/min/1.73 m^2^. Thus, this study was referenced as ARC for the sake of completeness.

## 4. Discussion

This review highlights the scarcity of evidence regarding optimal dose-regimens for NBLA in some of the special scenarios encountered among ICU patients. Variations within the studied settings and PK/PD-used targets across the included studies limited the overall generalizability of the findings. This is in line with earlier assessments that highlighted the lack of adequate information when conventional β-lactams were utilized among similar special scenarios [21,44]. Given the altered physiology/pathology of critically ill patients, standard dose regimens may be insufficient for NBLA and may increase the risk of therapeutic failure and/or resistance development. Such conclusion can be inferred from the pool of evidence pertaining to conventional β-lactams, where different antimicrobials were reported to be associated with altered PK when utilized among critically ill patients, and had suboptimal exposures when utilized at the usual manufacturer recommended dosing. Readers are encouraged to refer to the previous published literature for more in-depth information [17,25,91,92]

Although the “one size fits all” approach has been well-described to be inappropriate across such critically ill population, more studies are still required to properly adopt patient-centered treatment approaches [93]. The importance of achieving PK/PD targets in critically ill patients has recently been emphasized, considering the increasing microbial resistance, limited treatment alternatives, and severity of illnesses [94]. Various factors should be considered when administering such NBLA among critically ill patients with special circumstances, including critical-illness-related PK alterations, the physiochemical properties of the NBLA, the site of infection/MIC of invading pathogen, and the type/modality of extracorporeal support required [14,15]. However, caution should still be practiced when considering these factors. For instance, hydrophilic drugs with limited protein binding were previously reported to be minimally subjected to ECMO-mediated variations [52,95] Thus, one would extrapolate such minimal effects of ECMO to NBLA’s PK given their similar physiochemical properties and support such extrapolation by the reported studies highlighted in the Section 3 above. However, recent ex-vivo ECMO models have shown substantial removal of various NBLAs (e.g., C/T [96] and MEV [97]. Despite the limitations of such ex-vivo models in terms of correlation with clinical settings (i.e., single dosing administrations, no effects of human metabolism/clearance, different oxygenators used, effects of different primed fluids, etc.), they reveal commonly encountered variations seen in practice, and thus highlight the need for more clinical research to clarify such heterogeneity. 

Similarly, the effect of obesity on the PK of different antimicrobials has been a topic of debate. Despite being associated with alterations in different physiological parameters, including increased cardiac output and renal blood flow [44,47,48], different studies questioned the clinical significance in terms of required dosage adjustments. For instance, in a population PK study conducted by Alobaid and colleagues [48], obesity was associated with an altered central volume of the distribution of meropenem, yet such alteration did not translate into an altered probability of attaining PD targets. Rather, in their analysis, CrCL was found to be the significant covariate affecting PTA. Similar conclusions were drawn for other conventional β-lactams, too [49,98]. This might be in line with what we found in our review. Among the two reports of morbid obesity included in this review, both patients had altered renal functionalities (ARC in the case of ceftobiprole [50], and anuric requiring CVVHDF in the case of C/T [51]). In both cases, modifications targeted toward the altered renal functionality resulted in adequate exposures of the studied NBLA, without the requirement of further adjustments targeted towards BMI. 

Renal functionality has been well documented to derive major changes in the altered PK of different antimicrobials, including β-lactams. ARC has been suggested to be associated with subtherapeutic plasma concentrations of conventional β-lactams, along with increased rates of therapeutic failure when utilized at the standard dosing regimens [59,60,99,100]. Such studies have proposed increased dosing requirements and/or modified dosing administrations (i.e., prolonged infusions) to ensure attaining therapeutic targets. Comparing the results of conventional β-lactams’ modified dosing requirements in the setting of ARC to the studies of NBLA included in this review provide similar conclusions. Based on the limited available evidence from the included studies, modified administrations seemed to be required to achieve required PTA of different NBLAs. On the other extreme, Gatti and Pea [14,15] suggested four main factors to be considered when designing an appropriate regimen in the setting of CRRT. These included PK features/physiochemical properties of the utilized NBLA, RRT modality/setting/filter type, infection site/associated MIC of the pathogen, and critical illness related altered PK. Similar concepts are still applicable to the setting of PIRRT.

Considering the aforementioned variabilities associated with the four included special scenarios, TDM-based dosing coupled with modified dosing administrations (i.e., increased dosing and/or prolonged infusions) might still be warranted. Although a recent review questioned the role of comprehensive β-lactam TDM among critically ill patients, authors acknowledged the role of targeted TDM among special scenarios(including ARC, RRT, ECMO and obesity), where the benefits of preventing over or underexposure would be expected [101]. Nevertheless, keeping in mind that TDM is still not widely available across many centers worldwide, and until more robust data are available, one might consider utilizing actual creatinine clearance (e.g., using 12 h urine collection) to design an initial dosing regimen, especially in the settings of obesity and ECMO, where CrCL seemed to be the main determinant of PTA of NBLAs based on the available evidence included in this review.

### Limitations

The findings of this review should be interpreted with caution. Although this is not a systematic review, we sought to include most of the relevant studies reported. Furthermore, most of the retrieved studies included small patient populations and were of low quality (i.e., case reports/series). Finally, remarkable heterogeneity was detected in the targeted %ƒT > MIC across different reports/studies (highlighted in Table 2). Therefore, extrapolating conclusions should be thoughtfully considered when different PK/PD indices are clinically targeted.

## 5. Conclusions

Alterations in PK are often encountered in critically ill adult patients, and can result in suboptimal %ƒT > MIC attainments and potential therapeutic failures. This can be further complicated by many of the commonly encountered circumstances during critical illness, including obesity, ECMO, and extreme renal functionalities (ARC/RRT). The available literature, although confined, highlights the limitations of current dosing strategies in such settings. Different studies and reports have suggested modified dosing approaches (such as increased dosing and/or prolonged infusions) to optimize NBLA dosing across ICU-encountered scenarios, yet evidence has been limited by small sample sizes and the low quality of the studies. More robust, well-designed, studies are still required to determine the optimal dosing strategies of NBLA for such patient populations, and thus aid in improving clinical outcomes. Until more robust data are available, the use of TDM two guide therapy in such specialized scenarios might still be warranted.

## Figures and Tables

**Table 1:** Eligibility criteria for the included studies.

Population	1. Critically ill adult patients (ICU admissions) 2. Having any of the special scenarios (i.e., obesity, augmented renal clearance (ARC), extracorporeal interventions for life support (i.e., ECMO, prolonged intermittent renal replacement therapy (PIRRT), or continuous renal replacement therapy (CRRT))
Intervention	Receiving novel β-lactam antibiotics of interest (including Ceftolozane/Tazobactam, Ceftazidime/Avibactam, Cefiderocol, Ceftobiprole, Imipenem/Relebactam and Meropenem/Vaborbactam) with reported dose being used
Comparator	None
Outcomes	1. Reported PK profile of different novel β-lactam antibiotics under different scenarios studied 2. Reported PK/PD target attained with different doses used +/− clinical outcomes associated with use of drug therapy under studied circumstances
Study design	PK studies (including phase 1 trials), population pharmacokinetic analyses (PopPK), PD studies, case-reports/case series, or clinical trials if PK/PD characteristics were reported

**Table 3:** Suggested initial dosing of novel β-lactam antibiotics based on aimed PK/PD targets among critically ill patients with special scenarios.

Novel β-Lactam Antibiotic	Standard Dose (Normal Kidney Function)	Aimed PK/PD Target (In-Vitro/Murine vs. 100% *f*T > MIC)	ARC	RRT	ECMO	Obesity
**Ceftolozane/Tazobactam**	1.5–3 g Q8h (over 1–3 h)	**C: 40% *f*T > MIC** **T: 20% *f*T > C_T:1 mg/L_**	1.5 g LD then 4.5 g/24 h C.I (*if CI is not feasible, can consider 1.5 g Q8h for MIC ≤ 4 mg/L)*	**CVVHDF** 3 g LD then 0.75 g Q8h (over 1 h) *for MIC ≤ 4 mg/L*	NR ^‡^	NR ^‡^
				**CVVH/CVVHD/PIRRT** NA		
		**100% *f*T > MIC**	1.5 g LD then 4.5 g/24 h C.I *(if CI is not feasible, can consider 3 g Q8h for MIC ≤ 4 mg/L)*	**CVVHDF** 3 g LD then 0.75 g Q8h (over 1 h) *for MIC ≤ 4 mg/L*	3 g Q8h (over 1 h)	3 g Q8h (over 1 h)
				**PIRRT** *Off dialysis days:*LD 0.75 g, then 0.15 g Q8h (over 1 h) *Dialysis days:*0.75 g Q12h (over 1 h)		
				**CVVH/CVVHD** NA		
**Ceftazidime/Avibactam**	2.5 g Q8h (over 2 h)	**CAZ: 50% *f*T > MIC** **AVI: 50% *f*T > C_T:1 mg/L_**	2.5 g Q8h (over 2 h) *for MIC ≤ 16 mg/L*	**CVVH/CVVHD/CVVHDF/PIRRT** NA	NA	NA
		**100% *f*T > MIC**	NR	**CVVHDF**: 2.5 g Q8h (over 2 h) *for MIC ≤ 8 mg/L*	NA	NA
				**CVVH/CVVHD/PIRRT** NA		
**Cefiderocol**	2 g Q8h (over 3 h)	**75% *f*T > MIC**	2 g Q6h (over 3 h)	**CVVH/CVVHD/CVVHDF** **All doses infused over 3 hr***(for MIC ≤ 4 mg/L)* -*Effluent flow rate ≤ 2 L/h* → 1.5 g Q12h -*Effluent flow rate 2.1–3 L/h* → 2 g Q12h -*Effluent flow rate 3.1–4 L/h*→ 1.5 g Q8h -*Effluent flow rate ≥ 4.1 L/h*→ 2 g Q8h	NR ^‡^	NA
				**PIRRT** NA		
		**100% *f*T > MIC**	NA	**CVVHDF** 2 g Q8h (over 3 h) *for MIC ≤ 1 mg/L* **CVVH/CVVHD/PIRRT** NR	2 g Q8h (over 3 h) *for MIC ≤ 2 mg/L*	NA
**Ceftobiprole**	0.5 g Q8h (over 2 h)	**25–40% *f*T > MIC**	NR ^‡^	**CVVH/CVVHD/CVVHDF/PIRRT** NA	NA	NR ^‡^
		**100% *f*T > MIC**	0.5 g Q8h (**increase infusion rate to over 4 h**) *for MIC ≤ 4 mg/L*	**CVVHDF**: 0.25 g Q12h (over 2 h) *for MIC ≤ 2 mg/L*	NA	0.5 g Q8h (**Increase infusion rate to over 4 h**) *for MIC ≤ 2 mg/L*
				**CVVH/CVVHD/PIRRT** NA		
**Imipenem/Relebactam**	1.25 g Q6 (over 0.5 h)	**IMI: 40% *f*T > MIC** **REL: fAUC/MIC = 7.5**	1.25 g Q6h (over 0.5 h) *for MIC ≤ 2 mg/L*	**CVVH/CVVHD/CVVHDF/PIRRT** NA	NA	NA
		**100% *f*T > MIC**	NR		NA	NA
**Meropenem/Vaborbactam**	2–4 g Q8 (over 3 h)	**ME: 45% *f*T > MIC** **V: fAUC/MIC≥ 18–24**	NA	**CVVH/CVVHD/CVVHDF/PIRRT** NR ^‡^	NA	NA
		**100% *f*T > MIC**	NA	**CVVHD** 2 g Q8h (over 3 h) *for MIC ≤ 4 mg/L*	NA	NA
				**CVVH/CVVHDF/PIRRT** NA		

**IMI/REL**: imipenem/relebactam, **CAZ/AVI**: ceftazidime/avibactam, **C/T**: ceftolozane/tazobactam, **MEV**: meropenem/vaborbactam, **LD**: loading dose, **MIC**: minimum inhibitory concentration, **ARC**: augmented *renal* clearance, **MIC**: minimum inhibitory concentration, **RRT**: renal replacement therapy, **ECMO**: extracorporeal membrane oxygenation, **CVVH**: continuous veno-venous hemofiltration, **CVVHD**: continuous venovenous hemodialysis, **CVVHDF**: continuous venovenous hemodiafiltration, **PIRRT**: prolonged intermittent renal replacement therapy, **NR**: not reported as a specific target of the included studies, **NA**: not available/no studies found, **C.I**: continuous infusion. ‡ Dosing adaptation from 100%*f*T > MIC target can be considered. However, the effects of higher exposures or the need of decreased dosing requirements have not be studied/reported for this conventional PK/PD index among critically ill patients presenting with this special scenario.

## Supplementary Materials

The following supporting information can be downloaded at: https://www.mdpi.com/article/10.3390/jcm11236898/s1, Table S1: Pharmacokinetics and murine/in vitro-derived Pharmacodynamic targets of novel β-lactams.

## Abbreviations

AKI	acute kidney injury
ARC	augmented renal clearance
AUC	area under the concentration time curve
BLBLI	β-lactam/β-lactamase inhibitors
BLI	β-lactamase inhibitors
BMI	body mass index
CAZ/AVI	ceftazidime/avibactam
CFR	cumulative fraction of response
CFU	colony-forming units
C.I	continuous infusion
CL	clearance
Cmax	maximum concentration
Cmin	minimal concentration
CrCL	creatinine clearance
CRRT	continuous renal replacement therapy
CT	threshold of concentration
C/T	ceftolozane/tazobactam
CVVH	continuous venovenous hemofiltration
CVVHD	continuous venovenous hemodialysis
CVVHDF	continuous venovenous hemodiafiltration
ECMO	extracorporeal membrane oxygenation
E.I	extended infusion
g	gram
h	hour
ICUs	intensive care units
IMI/REL	imipenem/relebactam
LD	loading dose
MDR	multidrug-resistant
MEV	meropenem/vaborbactam
MIC	minimum inhibitory concentration
MRSE	methicillin-resistant *S. epidermidis*
PICO	population, intervention, comparator, outcome
PIRRT	prolonged intermittent renal replacement therapy
PK/PD	pharmacokinetic/pharmacodynamic
PsA	Pseudomonas aeruginosa
PTA	probability of target attainment
Qxh	every x hours
RRT	renal replacement therapy
TDM	therapeutic drug monitoring
VA-ECMO	venoarterial ECMO
Vd	volume of distribution
VV-ECMO	venovenous ECMO
%ƒT > MIC	percentage of free drug concentration above MIC
