# Supplementary material for: Pharmacokinetics, Pharmacodynamics, and Dosing Considerations of Novel β-Lactams and β-Lactam/β-Lactamase Inhibitors in Critically Ill Adult Patients: Focus on Obesity, Augmented Renal Clearance, Renal Replacement Therapies, and Extracorporeal Membrane Oxygenation
Source: J Clin Med. 2022 Nov 22;11(23):6898. doi: 10.3390/jcm11236898 (PMC9738279; doi:10.3390/jcm11236898)
Supplement: Supplementary file 1 [file jcm-11-06898-s001.zip › jcm-1962234-supplementary.pdf]

## Table S1: Pharmacokinetics and murine/in vitro-derived Pharmacodynamic targets of novel β-lactams

| Novel β-lactam antibiotic | Pharmacokinetic properties (General Population) | | | | | Pharmacodynamic targets based on Preclinical/Clinical data | | | | | | | |
|---|---|---|---|---|---|---|---|---|---|---|---|---|---|
| | VD (L) | t1/2 (h) | %protein bound | Renal CL | $AUC_{ELF}$/ plasma | Model | organism | Stasis | 1-log kill | 2-log kill | Used parameter | Target from previous study [1] | Additional comments |
| Cefiderocol | 18 | 2-3 | 40-60 | 90-98% | 0.10-0.24 | Murine thigh (neutropenic)[2] | *Enterobacterales* | 62.5%±27.4% | 73.3%±23.3% | NR | %ƒT>MIC | 75% ƒT>MIC | Overall, 1-log10 killing is achieved with ~60% ƒT>MIC against wild-type gram-negatives, and ~85% against carbapenemase producing gram-negatives |
| | | | | | | | *P. aeruginosa* | 63.0%±15.5% | 72.2%±21.4% | NR | | | |
| | | | | | | Murine lung (neutropenic)[2] | *Enterobacterales* | NR | 64.4%±22.5% | NR | | | |
| | | | | | | | *P. aeruginosa* | NR | 70.3%±9.0% | NR | | | |
| | | | | | | | *A. baumannii* | NR | 88.1%±3.4% | NR | | | |
| | | | | | | | *S. maltophilia* | NR | 53.9%±18.1% | NR | | | |
| | | | | | | Murine thigh and lung (neutropenic) [3] | *Enterobacterales*<br>*P. aeruginosa*<br>*A. baumannii*<br>*S. maltophilia* | 40% -70% | 55% to 88% | NR | | | |
| Ceftazidime/ Avibactam | 17.0/ 22.2 | 2.7 | 7-10 | 72-87% | 0.26-0.35 | Neutropenic mouse lung infection[4] | *Enterobacterales* | 30% | NR | 50% | %ƒT>MIC | **Ceftazidime** 50% ƒT>MIC | Some reported using lower targets of 40-45% ƒT>MIC [7] |
| | | | | | | Neutropenic murine thigh[5] | *P. aeruginosa* | 40% | NR | NR | | | |
| | | | | | | Murine thigh[6] | *P. aeruginosa* | 19.4% | 20.6% | NR | %ƒT>$C_{T=1\ mg/L}$ | **Avibactam** 50% ƒT>$C_{T=1\ mg/L}$ | |
| | | | | | | Murine lung[6] | *P. aeruginosa* | 31% | 46.9% | NR | | | |
| Ceftolozane/ Tazobactam | 13.5/ 18.2 | 3.1 | 16-30 | 62-84% | 0.50-0.62 | Neutropenic murine thigh[8] | *E. coli*<br>*K. pneumoniae* | 26.3% ± 2.1% | 31.6 ± 1.65 | NR | T>MIC | **Ceftolozane** 40 % ƒT>MIC | **Ceftolozane**:<br>For Enterobacterales with ESBLs, stasis and 1-log kill were observed at T>MIC values of ~30% and 35% [8]. However, higher targets were still reported. Thus 40%ƒT>MIC is commonly targeted<br>**Avibactam**<br>Commonly used targets are either 20% ƒT≥1 mg/L[12] 50% ƒT ≥2mg/L[13] |
| | | | | | | | *P. aeruginosa* | 24% ± 3.3% | 31.5 ± 3.9 | NR | | | |
| | | | | | | Murine thigh[8] | *P. aeruginosa* | 31.2% ± 6.9% | 39.4 ± 7.5% | 42.0±8.1 1 | | | |
| | | | | | | | *S. pneumoniae* | 18.1%± 4.52% | 23.8%±3.75% | 26.7%±0. 94% | | | |
| | | | | | | In vitro chemostat[9] | *E. coli* | 35% | 50% | 70% | %ƒT>$C_T$ | **Avibactam** 20% ƒT>$C_{T=1\ mg/L}$ | |
| | | | | | | In vitro chemostat[10] | *E. coli*<br>*K. pneumonia* | 65.9% | 77.3% | 90.2% | %ƒT>$C_{T=0.5mg/L}$ | | |
| | | | | | | Murine thigh[11] | *E. coli*<br>*K. pneumonia* | 28.2% | 44.4% | NR | %ƒT>$C_{T=0.5mg/L}$ | | |
| Imipenem/ Relebactam | 24.3/ 19.0 | 1.2 | 20-22 | 52-92% | 0.54-0.55 | Neutropenic Murine Thigh[14] | *P. aeruginosa* | NR | 40% | NR | %ƒT>MIC | **Imipenem** 40% ƒT>MIC | |
| | | | | | | Murine thigh[15] | *K. pneumonia*<br>*P. aeruginosa* | 26 | NR | NR | ƒAUC | **Relebactam** ƒAUC/MIC = 7.5 | |
| | | | | | | Neutropenic Murine Thigh[14] | *P. aeruginosa* | 3.3 | 4.3 | 7.0 | ƒAUC/MIC | | |
| Meropenem/ Vaborbactam | 20.2/ 18.6 | 2.3 | 2-33 | 74% | 0.63-0.79 | Neutropenic Mouse Thigh[16] | *A. baumannii* | 7.3%– 24.2% | 14.5%–36.9% | 26%– 53.2% | %ƒT>MIC | **Meropenem** 45% ƒT>MIC | Animal infectivity models suggest ≥40%ƒT >MIC are required to maximize |
| | | | | | | Neutropenic murine thigh[17] | *A. baumannii* | 24% | 33% | 48% | | | |

| | | | | | | | | | | | | | |
|---|---|---|---|---|---|---|---|---|---|---|---|---|---|
| | | | | | | Neutropenic thigh[3] | Gram-negatives | 30% | 35% | 45% | | | bactericidal activity with meropenem[19] |
| | | | | | | In vitro hollow fiber[18] | *K. pneumonia* *Enterobacter cloacae* | 12 | 18 | 25 | ƒAUC/MIC | **Vaborbactam** ƒAUC/MIC≥18-24 | |
| | | | | | | Murine thigh[18] | *K. pneumonia* *Enterobacter cloacae* *E. coli* | 9 | 38 | 220 | | | |
| **Ceftobiprole** | 21.7 | 3.1 | 16 | 83% | 0.68[20] | Neutropenic thigh[21] | *Enterobacter* | 40.8% ± 3.8% | NR | 64.5% ± 25.1% | %T> MIC | 30 - 50%ƒT > MIC | Different ranges have been reported by different studies based on the in-vivo model studied, infecting pathogen, and associated MIC |
| | | | | | | | *S. aureus* | 21.1%± 3.9% | NR | 29.3%± 4.6% | | | |
| | | | | | | | *S. pneumoniae* | 18.8% ± 2.7% | NR | 25.8% ± 4.8% | | | |

***P. aeruginosa:*** Pseudomonas aeruginosa, ***A. baumannii:*** Acinetobacter baumannii, ***S. maltophilia:*** Stenotrophomonas maltophilia, ***E. coli:*** Escherichia coli, ***K. pneumoniae:*** Klebsiella pneumoniae, ***S. aureus***: Staphylococcus aureus, ***S. pneumoniae****: Streptococcus pneumoniae,* Vd: volume of distribution, t1/2: half-life, CL: clearance, AUC: area under the concentration time curve, ELF: Epithelial lining fluid, NR: not reported, MIC: minimum inhibitory concentration, %ƒT>MIC: percentage of free drug concentration above MIC, $C_T$: threshold of concentration

## References:


1- Gatti M, Pea F. Pharmacokinetic/pharmacodynamic target attainment in critically ill renal patients on antimicrobial usage: focus on novel beta-lactams and beta lactams/beta-lactamase inhibitors. Expert Rev Clin Pharmacol. 2021 May;14(5):583-599.
2- Nakamura R, Ito-Horiyama T, Takemura M, Toba S, Matsumoto S, Ikehara T, Tsuji M, Sato T, Yamano Y. In Vivo Pharmacodynamic Study of Cefiderocol, a Novel Parenteral Siderophore Cephalosporin, in Murine Thigh and Lung Infection Models. Antimicrob Agents Chemother. 2019 Aug 23;63(9):e02031-18.
3- Berry AV, Kuti JL. Pharmacodynamic Thresholds for Beta-Lactam Antibiotics: A Story of Mouse Versus Man. Front Pharmacol. 2022 Mar 18;13:833189.
4- Andes D, Craig W. 2002. Animal model pharmacokinetics and pharmacodynamics: a critical review. *Int J Antimicrob Agents* 19:261–268.
5- Craig WA. Basic pharmacodynamics of antibacterials with clinical applications to the use of beta-lactams, glycopeptides, and linezolid. Infect Dis Clin North Am. 2003 Sep;17(3):479-501
6- Berkhout J, Melchers MJ, van Mil AC, et al. Pharmacodynamics of ceftazidime and avibactam in neutropenic mice with thigh or lung infection. Antimicrob Agents Chemother 2015;60:368–75.
7- Fantin B, Leggett J, Ebert S, Craig WA. Correlation between in vitro and in vivo activity of antimicrobial agents against gram-negative bacilli in a murine infection model. Antimicrob Agents Chemother. 1991 Jul;35(7):1413-22.
8- Craig WA, Andes DR. In vivo activities of ceftolozane, a new cephalosporin, with and without tazobactam against Pseudomonas aeruginosa and Enterobacteriaceae, including strains with extended-spectrum beta-lactamases, in the thighs of neutropenic mice. Antimicrob Agents Chemother. 2013;57(4):1577–1582.
9- VanScoy B, Mendes RE, Nicasio AM, et al. Pharmacokinetics pharmacodynamics of tazobactam in combination with ceftolozane in an in vitro infection model. Antimicrob Agents Chemother 2013;57:2809–14.
10- Vanscoy B, Mendes RE, McCauley J, et al. Pharmacological basis of beta-lactamase inhibitor therapeutics: tazobactam in combination with ceftolozane. Antimicrob Agents Chemother 2013;57:5924–30.
11- Melchers MJ, Mavridou E, van Mil AC, Lagarde C, Mouton JW. Pharmacodynamics of ceftolozane combined with tazobactam against Enterobacteriaceae in a neutropenic mouse thigh model. Antimicrob Agents Chemother 2016;60:7272–9.
12- Chaijamorn W, Shaw AR, Lewis SJ, Mueller BA. Ex vivo Ceftolozane/Tazobactam Clearance during Continuous Renal Replacement Therapy. Blood Purif. 2017;44(1):16-23.
13- Xiao AJ, Caro L, Popejoy MW, Huntington JA, Kullar R. PK/PD Target Attainment With Ceftolozane/Tazobactam Using Monte Carlo Simulation in Patients With Various Degrees of Renal Function, Including Augmented Renal Clearance and End-Stage Renal Disease. Infect Dis Ther. 2017 Mar;6(1):137-148.
14- Patel, M., Daryani, N. M., Fen, H. P., Hilbert, D. W., Melchers, M. J., Mavridou, E., et al. "Imipenem/Relebactam Pharmacokinetic/ Pharmacodynamic Analyses from an In Vivo Neutropenic Murine Thigh Infection Model (Abstract 1693)," in Proceeding of the 30th ECCMID, Paris, France, April 2020.
15- Mavridou E, Melchers RJ, van Mil AC, Mangin E, Motyl MR, Mouton JW. Pharmacodynamics of imipenem in combination with beta-lactamase inhibitor MK7655 in a murine thigh model. Antimicrob Agents Chemother 2015;59:790–5.
16- Sabet M, Tarazi Z, Griffith DC. Pharmacodynamics of Meropenem against Acinetobacter baumannii in a Neutropenic Mouse Thigh Infection Model. Antimicrob Agents Chemother. 2020 Mar 24;64(4):e02388-19.
17- Macvane SH, Crandon JL, Nicolau DP. Characterizing in vivo pharmacodynamics of carbapenems against Acinetobacter baumannii in a murine thigh infection model to support breakpoint determinations. Antimicrob Agents Chemother. 2014;58(1):599-601.
18- Griffith DC, Sabet M, Tarazi Z, Lomovskaya O, Dudley MN. Pharmacokinetics/Pharmacodynamics of Vaborbactam, a Novel Beta-Lactamase Inhibitor, in Combination with Meropenem. Antimicrob Agents Chemother. 2018 Dec 21;63(1):e01659-18.
19- Patel TS, Pogue JM, Mills JP et al. Meropenem–vaborbactam: a new weapon in the war against infections due to resistant Gram-negative bacteria. Future Microbiol. 2018 Jul;13(9):971-983.
20- Rodvold KA, Nicolau DP, Lodise TP, et al. Identifying exposure targets for treatment of staphylococcal pneumonia with ceftobiprole. Antimicrob Agents Chemother. 2009;53(8):3294-3301.
21- Craig WA, Andes DR. In vivo pharmacodynamics of ceftobiprole against multiple bacterial pathogens in murine thigh and lung infection models. Antimicrob Agents Chemother. 2008 Oct;52(10):3492-6.
